# Identification of germline cancer predisposition variants in pediatric sarcoma patients from somatic tumor testing

**DOI:** 10.1038/s41598-023-29982-2

**Published:** 2023-02-20

**Authors:** Piedad Alba-Pavón, Lide Alaña, Miriam Gutierrez-Jimeno, Susana García-Obregón, Teresa Imízcoz, Elena Panizo, Paula González-Urdiales, Aizpea Echebarria-Barona, Ricardo Lopez Almaraz, Laura Zaldumbide, Itziar Astigarraga, Ana Patiño-García, Olatz Villate

**Affiliations:** 1 Pediatric Oncology Group, Biocruces Bizkaia Health Research Institute, Barakaldo, Spain; 2 Department of Pediatrics, Clinica Universidad de Navarra, Pamplona, Spain; 3 Department of Physiology, Faculty of Medicine and Nursing, University of the Basque Country, UPV/EHU, Leioa, Spain; 4 CIMA LAB Diagnostics, University of Navarra, Pamplona, Spain; 5 Pediatrics Department, Hospital Universitario de Cruces, Osakidetza, Barakaldo, Spain; 6 Department of Pathology, Hospital Universitario de Cruces, Osakidetza, Barakaldo, Spain; 7 Pediatrics Department, Faculty of Medicine and Nursing, University of the Basque Country, UPV/EHU, Leioa, Spain; 8 Center for Applied Medical Research and IdiSNA, Solid Tumor Program, CIMA, Pamplona, Spain

**Keywords:** Cancer genetics, Paediatric cancer, Sarcoma, Next-generation sequencing, Paediatric research, Sarcoma

## Abstract

Genetic predisposition is an important risk factor for cancer in children and adolescents but detailed associations of individual genetic mutations to childhood cancer are still under intense investigation. Among pediatric cancers, sarcomas can arise in the setting of cancer predisposition syndromes. The association of sarcomas with these syndromes is often missed, due to the rarity and heterogeneity of sarcomas and the limited search of cancer genetic syndromes. This study included 43 pediatric and young adult patients with different sarcoma subtypes. Tumor profiling was undertaken using the Oncomine Childhood Cancer Research Assay (Thermo Fisher Scientific). Sequencing results were reviewed for potential germline alterations in clinically relevant genes associated with cancer predisposition syndromes. Jongmans´ criteria were taken into consideration for the patient selection. Fifteen patients were selected as having potential pathogenic germline variants due to tumor sequencing that identified variants in the following genes: *CDKN2A*, *NF1, NF2, RB1*, *SMARCA4*, *SMARCB1* and *TP53*. The variants found in *NF1* and *CDKN2A* in two different patients were detected in the germline, confirming the diagnosis of a cancer predisposition syndrome. We have shown that the results of somatic testing can be used to identify those at risk of an underlying cancer predisposition syndrome.

## Introduction

Genetic predisposition is an important risk factor for cancer in children and adolescents^1^. Recent studies indicate that a considerable proportion of pediatric cancers are related to germline mutations in cancer predisposition genes^2,3^.

The identification of genes related to the hereditary predisposition to cancer in children and adolescents with tumors, their frequency of mutation, the ethical implications of their testing and the importance for family counseling, are still fields under intense investigation. Recently, 751 patients with solid tumors underwent prospective matched tumor–normal DNA sequencing with downstream clinical use and the results showed that 18% (138/751) of individuals had one or more germline pathogenic or likely pathogenic variants including variants in low-, moderate- and high-penetrance dominant or recessive genes^3^. Another large study of exome and genome sequencing of 1120 children and adolescents with all types of tumors identified inheritable mutations in 8.5% of cancer patients, however only 40% of patients with pathogenic or probably pathogenic germline mutations had a family history of cancer^2^.

According to several studies, approximately 10% of pediatric cancer patients are considered to have a germline mutation^4,5^. For some patients, the prevalence of mutations may be higher, as it has been observed in children with choroid plexus carcinoma of which 50% have germline mutations in *TP53*^6,7^ or malignant rhabdoid tumors, of which 25–35% have mutations in *SMARCB1*^8,9^. The identification of an inherited genetic variant in a pediatric patient allows physicians to better guide the future management of the patients, as well as to provide genetic counseling to the patients and their families.

Among pediatric cancers, sarcomas often occur sporadically, but they can also arise in the setting of heritable cancer predisposition syndromes. Sarcomas are neoplasms of mesenchymal origin that comprise only 1% of adult malignancies, but a significantly greater proportion (15%) of childhood cancers^10^. Pediatric sarcomas are largely divided between those that arise from bone and soft tissues. Nevertheless, they are very heterogeneous, comprising more than 70 distinct histological subtypes with differences in genetic complexity and driver molecular aberrations^11^. In addition, several sarcoma subtypes present specific genomic alterations, such as pathognomonic gene fusions. The association of particular sarcomas with various hereditary cancer predisposition syndromes adds even more complexity. These associations are often disregarded, given the rarity and diversity of sarcomas and the equivalent relative infrequency of cancer genetic syndromes.

Among sarcomas, the most common malignant bone tumors are osteosarcoma and Ewing sarcoma, while rhabdomyosarcoma is the most common soft tissue sarcoma. Approximately 10% of osteosarcomas are associated with genetic cancer predisposition mutations. Of note, Li Fraumeni (LFS) and hereditary retinoblastoma syndromes^12^ are often associated with osteosarcoma. In addition, a recent study identified a pathogenic or likely pathogenic cancer-susceptibility gene variant in 28% of patients with osteosarcoma. Furthermore, these variants were observed not only in *TP53* but also in genes not previously linked to osteosarcoma as *CDKN2A, ATRX, APC* or *MSH3*^13^. Interestingly, genetic predisposition syndromes are less frequent in patients with sarcomas carrying specific gene fusions (as Ewing sarcoma, alveolar rhabdomyosarcoma or synovial sarcoma). Several studies related to germline predisposition to Ewing sarcoma have focused on the identification of susceptibility loci from genome-wide association studies^14–16^. In addition, Ewing sarcoma has been recently associated with germline pathogenic variants in genes involved in DNA damage repair such as *FANCC*, *CHEK2*, *BRAC1* and *BRCA2*^17,18^. Numerous reports and studies of individual genetic disease cohorts highlight that children with genetic syndromes develop rhabdomyosarcoma more frequently than unaffected peers^19^. Genetic risk of rhabdomyosarcoma results from germline predisposition variants associated with a wide spectrum of cancer susceptibility syndromes^20^. These include LFS, hereditary retinoblastoma syndrome, Beckwith-Wiedemann syndrome and RASopathies such as Costello syndrome and neurofibromatosis type 1^21^. The majority of syndromic rhabdomyosarcomas have been described in those without the *PAX3/7::FOXO1* translocations^21^. Other soft tissue sarcomas have also been associated with a cancer predisposition syndrome such as neurofibromatosis type 1 associated to GIST (Gastrointestinal Stromal Tumors) and MPNST (Malignant Peripheral Nerve Sheath Tumors)^10^.

Bearing in mind the association between sarcomas and some predisposition syndromes, germline mutations should be analyzed in this group of tumors. However, the most commonly used NGS (next-generation sequencing) panels involve tumor-first sequencing and do not include germline testing. In a previous study, Klek et al. demonstrated in adult patients with a solid tumor malignancy that tumor sequencing could provide an opportunity to detect germline pathogenic variants. They showed that careful review of tumor sequencing data substantially increased the percentage of cancer patients in their cohort diagnosed with a hereditary cancer susceptibility^22^.

In this context, we hypothesized that reviewing tumor-first test results for potential germline alterations together with family history, tumor characteristics and patient data would increase the rate of germline pathogenic variant detection. In fact, detection of germline pathogenic variants plays an important role in clinical management of patients and families and emphasizes the importance of genetic counseling when these pathogenic variants are detected.

## Results

### Patient characteristics

Clinical characteristics of the patients are included in Table 1. A total of 43 patients were selected for the study. The median age at diagnosis was 12 years-old (range 0.6–30.8 years-old). Overall, 88.3% of patients were European and 62.8% were male. Osteosarcoma (41.9%), Ewing sarcoma (27.9%) and rhabdomyosarcoma (9.3%) are the most common cancer diagnoses. There is 20.9% of other sarcoma types.

**Table 1:** Clinical characteristics of 43 patients with sarcoma.

Characteristics	Number (%)
Median age at diagnosis (range)	12.0 (0.6–30.8)
Gender	
Male	27 (62.8)
Female	16 (37.2)
Ethnic origin	
European	38 (88.3)
Latino	2 (4.7)
African	2 (4.7)
South Asian	1 (2.3)
Classification of the sarcoma	
Osteosarcoma	18 (41.9)
Ewing sarcoma	12 (27.9)
Rhabdomyosarcoma	4 (9.3)
Other	9 (20.9)
Location of the primary tumor	
Lower extremity	23 (53.5)
Upper extremity	10 (23.3)
Trunk	6 (14.0)
Other	4 (9.3)
Disease stage at diagnosis	
Localized	32 (74.4)
Metastatic	11 (25.6)
Treatment regimen	
Chemotherapy	42 (97.7)
Surgery	37 (86.0)
Radiotherapy	25 (58.1)
Targeted therapy	6 (14.0)
Immunotherapy	7 (16.3)
Stem cell transplant	6 (14.0)
Relapse	
Yes	23 (53.5)
No	20 (46.5)
Current status	
Alive	30 (69.8)
Dead	13 (30.2)

### Tumor NGS-based identification of pathogenic variants and selection of patients with a potential germline variant

**Figure 1:**
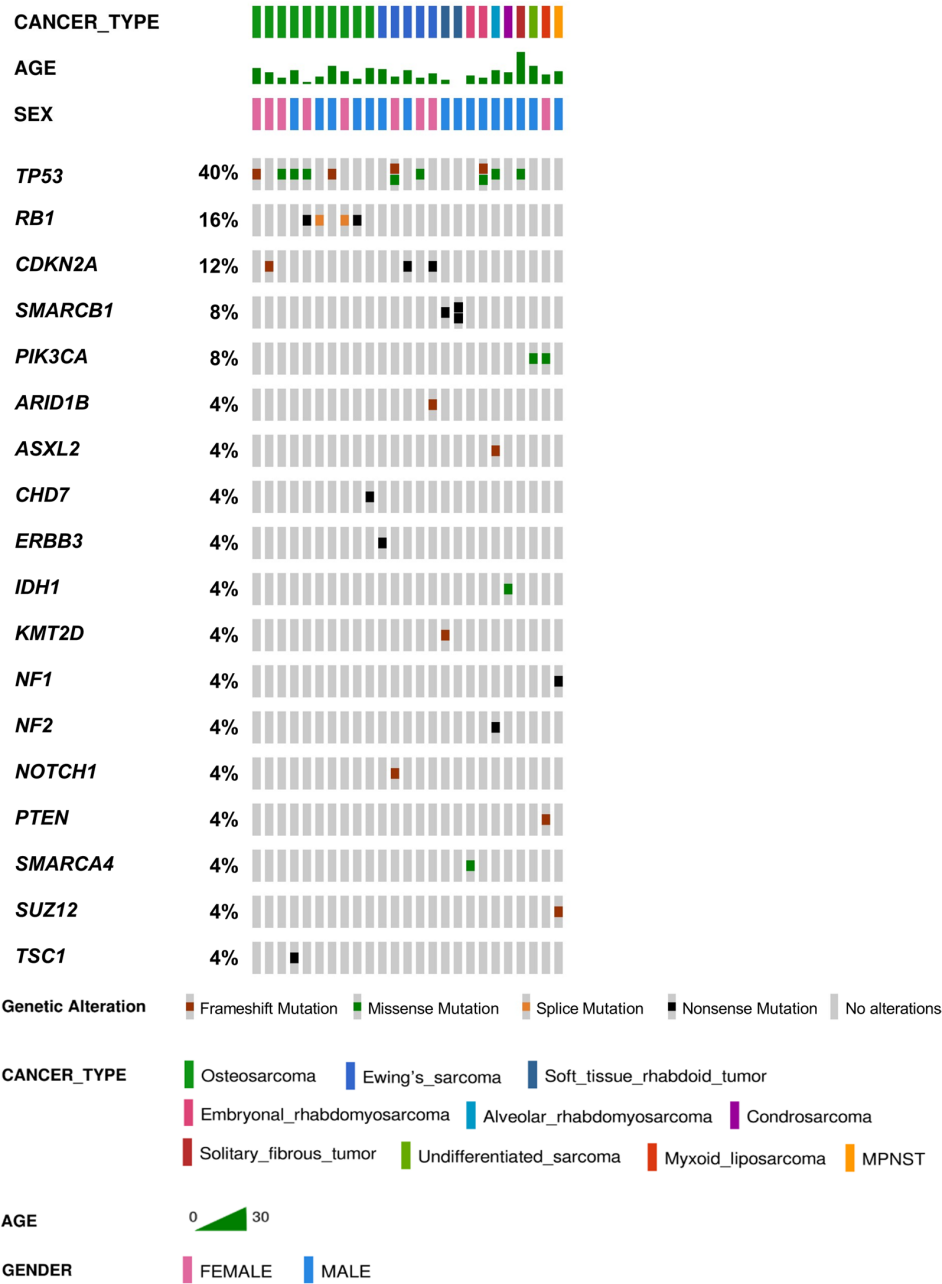
Pathogenic and likely pathogenic variants identified by somatic tumor sequencing in our cohort of 43 pediatric patients with sarcomas. Genetic alterations found by NGS in the different types of sarcoma are represented and classified according to the type of variant. The most mutated gene with this type of variants is *TP53* followed by *RB1*. Variants have been represented using OncoPrinter tool from cBioportal v5.1.6 (https://www.cbioportal.org/oncoprinter)^53^.

Samples from 39 primary tumors, 1 sample from costal metastases of osteosarcoma and 6 samples from different relapses from our cohort of 43 patients, were analyzed with the Oncomine Childhood Cancer Research Assay (Thermo Fisher Scientific). Results of all the potentially significant variants identified in our patients are included in Supplementary Table S1. After filtering, 79 variants were considered and classified as pathogenic (n = 31), likely pathogenic (n = 6) and variants of unknown significance (VUS) (n = 42). Pathogenic and likely pathogenic somatic variants found in our cohort are represented and classified according to the type of mutation in Fig. 1.

Twenty-eight of the 79 variants were found in genes associated with cancer predisposition syndromes (Supplementary Table S1) but only 23 were pathogenic or likely pathogenic. Three of these variants were discarded as they had a Variant Allele Frequency (VAF) less than 20% in tumor-sample sequencing (cases 6, 18 and 41). One of the variants was removed because it was detected in the metastasis but not in the primary tumor (case 25). Another case with a pathogenic variant in *RB1* was discarded because this patient showed the clinical features compatible with a Rothmund-Thompson syndrome and the diagnosis was confirmed by germline sequencing of the *RECQL4* gene (case 27)^23^. Therefore, from the 23 pathogenic or likely pathogenic variants selected for the study, five of them were removed due to the above reasons.

Finally, eighteen variants of fifteen patients were selected for further study. These variants were identified in genes as *TP53, CDKN2A, NF1, NF2, RB1, SMARCB1* and *SMARCA4* and they are highlighted in bold in Supplementary Table S1. In addition to the Jongmans´ criterion of genetic tumor analysis, medical records of these selected patients were examined searching for information related to tumor type, cancer family history, development of secondary malignancies, genomic tumor analysis, presence of congenital anomalies and toxicity due to cancer treatment. These criteria concerning family history, tumor characteristics and patient data used in this study are described in Table 2. Using this approach, we found two patients of our cohort with a family history of cancer. Two patients presented with congenital anomalies and other specific symptoms, one of them with excessive toxicity to cancer therapy. One patient had two malignant tumors and suffered from excessive toxicity to treatment. This patient developed a tubulopathy, a secondary cardiac dysfunction due to doxorubicin treatment and ototoxicity due to cisplatin treatment. In summary, only five patients had another additional Jongmans´ criterion. For the germline study, it was decided to analyze the 15 patients previously selected that represented a 34.9% of our cohort.

**Table 2:** Fulfilment of inherited genetic alteration criteria and associated genes in the 15 selected patients.

Patient	Tumor type	Family History	≥ 2 malignant tumors	Congenital Anomalies and other specific symptoms	Excessive toxicity to cancer treatments	Genetic tumor analysis
3	Ewing sarcoma	No	No	No	No	***TP53***
8	Ewing sarcoma	No	No	No	No	***CDKN2A***
13	Malignant peripheral nerve sheath tumor (MPNST)	No	No	Yes	No	***NF1***
14	Osteosarcoma	No	No	No	No	***TP53***
15	Osteosarcoma	No	No	No	No	***CDKN2A***
16	Osteosarcoma	No	No	Yes	Yes	***TP53***
17	Osteosarcoma	Yes	No	No	No	***TP53***
18	Osteosarcoma	No	Yes	No	Yes	***TP53***
20	Osteosarcoma	No	No	No	No	***RB1***
24	Chondroblastic osteosarcoma	No	No	No	No	***TP53***
32	Alveolar rhabdomyosarcoma	No	No	No	No	***TP53, NF2***
33	Embryonal rhabdomyosarcoma	No	No	No	No	***SMARCA4***
37	Solitary fibrous tumor	No	No	No	No	***TP53***
38	Soft tissue rhabdoid tumor	Yes	No	No	No	***SMARCB1***
39	Soft tissue rhabdoid tumor	No	No	No	No	***SMARCB1***

### Variants analyzed in germline

The germline candidate genes with pathogenic or likely pathogenic variants were *CDKN2A, NF1, NF2, RB1, SMARCA4, SMARCB1* and *TP53* (Table 3). *TP53* was the most frequently mutated gene in our patient cohort and mutations in this gene were found in the tumors of 8 subjects: five patients diagnosed with osteosarcoma, one patient with alveolar rhabdomyosarcoma, one patient with Ewing sarcoma and other with solitary fibrous tumor (Table 3). All somatic variants identified in *TP53* were classified as pathogenic.

**Table 3:** Description of the variants found in the tumors of selected patients and validated in germline.

GENE	PATHWAY	FUNCTION	SYNDROME	PATIENT	TUMOR	NT VARIANT	AA VARIANT	VAF^a^	VC^b^	GERMLINE^c^
*CDKN2A*	Cell cycle	Tumor suppresor	Familial Melanoma	15	Osteosarcoma	c.350del	p.(Leu117ArgfsTer29)	0.84	P	Yes
				8	Ewing Sarcoma	c.329G > A	p.(Trp110Ter)	0.50	P	No
*NF1*	GTPase activating protein	Tumor suppresor	Neurofibroma-tosis	13	MPNST	c.7152_7153insT	p.(Asn2385Ter)	0.5	P	Yes
*NF2*	Cytoskeletal Signaling	Tumor suppresor	Neurofibroma-tosis Type 2	32	Alveolar rhabdomyosarcoma*	c.586C > T	p.(Arg196Ter)	0.4	P	No
*RB1*	Cell cycle	Tumor suppresor	Heritable retinoblastoma	20	Osteosarcoma	c.1215 + 1G > A	p.?	0.65	P	No
*SMARCA4*	Chromatin remodeling	Tumor suppresor	Rhabdoid tumor predisposition	33	Embryonal Rhabdomyosarcoma	c.3694G > A	p.(Gly1232Ser)	0.2	P	-
*SMARCB1*	Chromatin remodeling	Tumor suppresor	Rhabdoid tumor predisposition	38	Soft tissue rhabdoid tumor	c.544C > T	p.(Gln182Ter)	0.95	LP	No
				39	Soft tissue rhabdoid tumor	c.472C > T	p.(Arg158Ter)	0.42	P	No
						c.118C > T	p.(Arg40Ter)	0.42	P	No
*TP53*	DNA damage	Tumor suppresor	Li Fraumeni	24	Chondroblastic osteosarcoma	c.618_624del	p.(Asp207GlufsTer38)	0.6	P	No
				14	Osteosarcoma	c.957_958insC	p.(Lys320GlnfsTer17)	0.6	P	No
				32	Alveolar rhabdomyosarcoma*	c.817C > T	p.(Arg273Cys)	0.6	P	No
				16	Osteosarcoma	c.818G > A	p.(Arg273His)	0.85	P	No
				17	Osteosarcoma	c.818G > A	p.(Arg273His)	0.64	P	No
				18	Osteosarcoma	c.475G > C	p.(Ala159Pro)	0.28	P	No
				3	Ewing sarcoma	c.404del	p.(Arg135SerfsTer73)	0.5	P	–
						c.817C > T	p.(Arg273Cys)	0.5		–
				37	Solitary fibrous tumor	c.713G > A	p.(Cys238Tyr)	0.4	P	–

^a^VAF: variant allele frequency.

^b^VC:Variant Classification based on ACMG guidelines. P: pathogenic; LP: likely pathogenic.

^c^Variant identified in germline. *Same patient.

All potential germline variants were in tumor suppressor genes. They were associated to different cancer predisposition syndromes and all of them had an autosomal-dominant inheritance: rhabdoid tumor predisposition syndrome (*SMARCA4* and *SMARCB1* genes), neurofibromatosis (*NF1, NF2*), familial melanoma (*CDKN2A*), hereditary retinoblastoma (*RB1*) and LFS (*TP53*). Variants were analyzed in DNA from blood samples of the selected patients to identify those also present in the germline. Three patients were excluded due to lack of sample. The variants identified in *SMARCB1* in two different patients with soft tissue rhabdoid tumors were not identified in the germline, thus confirming their somatic origin. The pathogenic variants in *TP53* were analyzed in six patients but they were not detected in the germline. The variants in *NF1* and *CDKN2A*, found in a patient with MPNST and a patient with osteosarcoma respectively, were detected in the germline, thus confirming the diagnosis of a cancer predisposition syndrome. Consistent with the diagnosis of neurofibromatosis, this patient also presented with multiple cafe au lait spots and subcutaneous neurofibromas as well as other deep lesions affecting the spinal nerves and abdominal region and had no family history of cancer. The patient with a *CDKN2A* germline pathogenic variant developed a fatal metastatic relapse during the osteosarcoma therapy.

## Discussion

Several cancer susceptibility genes are included in somatic panels so somatic testing could be an important source of clinically relevant germline findings, due to pathogenic variants in high penetrance genes. Our group has found that tumor sequencing identifies a substantial number of potentially germline variants and, after validation, 2 out of 15 selected patients (13%) carried germline variants and were thus diagnosed with cancer predisposition syndromes. These results are consistent with the findings of other studies showing that review of tumor-first NGS increased the discovery of germline pathogenic variants from tumor-first testing^22^.

We report that 34.9% of our patients with sarcomas had potentially pathogenic/likely pathogenic germline variants in cancer-susceptibility genes, with autosomal-dominant inheritance. According to several studies, approximately 10% of pediatric cancer patients are considered to have a germline mutation^4,5^. But an earlier study identified a pathogenic or likely pathogenic cancer-susceptibility gene variant in the 28% of patients with osteosarcoma^13^. We have confirmed the presence of the germline variant in the 13% of the patients we selected with a potentially germline variant. In this study, we confirmed previous observations of a high frequency of potentially germline *TP53* pathogenic/likely pathogenic variants in patients with osteosarcoma^13,24^. In our selected patients, we similarly identified pathogenic/likely pathogenic variants in *CDKN2A*, *RB1, NF1*, *NF2, SMARCA4* and *SMARCB1* genes*.*

It is important to consider the interplay between genetic ancestry and tumor mutational burden (TMB) as recently described^25^. TMB estimates from tumor-only panels substantially overclassify individuals into a TMB-high group due to false-positive germline variants, and this bias is particularly notable in patients with Asian/African ancestry. The study authors' suggestion to improve ancestral bias is the calibration of tumor-only TMB using paired tumor/normal TMB^25^. In our study there is a small proportion of non-European patients in which there may be a potential bias.

Our study identified a recently described as a new candidate sarcoma susceptibility gene, namely *CDKN2A*, which is worthy of further study. Germline variants in *CDKN2A* have been recently associated with osteosarcoma^13^. *CDKN2A* loss is an important somatic event in human osteosarcomas^24,26–28^ but until recently it had not been described in the germline. We found the germline *CDKN2A* c.350del (p.Leu117ArgfsTer29) pathogenic variant in a patient with osteosarcoma. This variant has been described in bladder cancer in adult patients but only at the somatic level^29^. Our patient with germline alteration in *CDKN2A* had pulmonary and bone metastases at diagnosis, relapsed during the treatment (bone and brain metastases) and eventually died due to disease progression.

*CDKN2A* mutations are responsible for the majority cases of hereditary melanoma. Additionally, melanoma risk is increased in mixed cancer syndromes caused by mutations in *PTEN, BRCA2, BRCA1, RB1* and *TP53*^30^. Germline mutations in *CDKN2A* increase the risk of melanoma by 65-fold^31^. In melanoma with *CDKN2A* germline mutations, there are usually somatic mutations in *BRAF* and *NRAS* genes, *NRAS* mutations being the most common ones^32,33^. In accordance with this observation, we also identified a somatic *BRAF* amplification in the same patient. It is interesting to mention that this tumor progressed with bone and brain metastases. In patients with osteosarcoma, relapses usually appear in other bones and pulmonary metastases, but brain metastases are not frequent^34^. Brain metastasis are frequent in patients with melanoma^35^.

Germline alterations in *CDKN2A* are most frequently associated with predisposition to melanoma and pancreatic cancer but some studies describe a susceptibility to neural system tumors, breast cancer, multiple myeloma, head and neck squamous cell carcinoma and sarcoma^36–38^. Therefore, the broad spectrum of cancer phenotypes potentially accompanying the germline alterations in *CDKN2A* suggests that it could be regarded as a candidate for tumor predisposition beyond melanoma and pancreatic cancer in clinical practice^30,39^. It would be interesting to consider *CDKN2A* germline alterations in patients with osteosarcoma that may be associated with a cancer predisposition syndrome, especially in those cases where LFS is ruled out.

In this study, we also found a pathogenic variant in *NF1* (c.7152_7153insT) in the tumor and in the germline of a patient with a MPNST. This variant had not been previously described in cancer^29^. MPNST is a non-rhabdomyosarcoma soft tissue sarcoma that arises from the peripheral nerve sheath tissue. In 50% of cases, they occur in the context of neurofibromatosis type I, characterized by loss of function mutations of the tumor suppressor neurofibromin (*NF1*)^40^. Prognosis is generally poor, with a high risk of relapse following multimodality therapy in early disease, low response rates of cytotoxic chemotherapy in advanced disease, and propensity for rapid disease progression and high mortality. It has been reported that about 50% of NF1 cases are due to de novo mutations^41^. Our patient had not a family history however, presented multiple cafe au lait spots and subcutaneous neurofibromas (Table 4), had three relapses during the treatment (pulmonary, axillary and extrapleural relapses) and died of disease progression. Since both patients with cancer predisposition syndromes in this cohort relapsed during treatment, this variable could be also considered to evaluate a potential cancer predisposition syndrome.

**Table 4:**
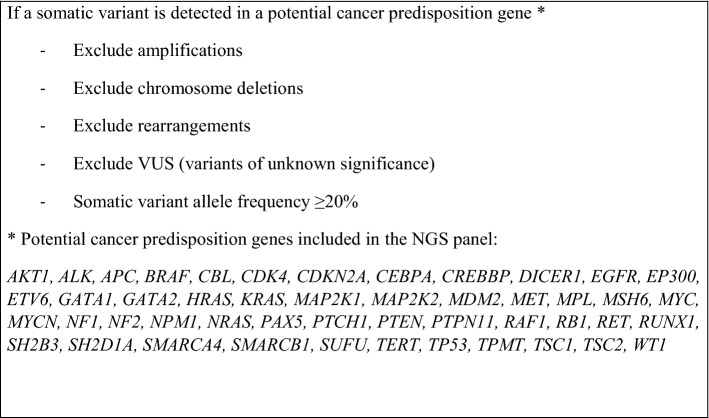
Rules applied to tumor-first NGS review to identify potential germline alterations.

Germline mutations of the *TP53* tumor suppressor gene cause LFS, an autosomal and dominant condition^42^. LFS is among the most aggressive cancer predisposition syndromes, characterized by a high rate and early-onset cancer risk. The classical definition of LFS requires an individual with a sarcoma diagnosed under the age of 45 who has at least one first-degree relative with a cancer of any kind diagnosed under the age of 45 and a third family member who is either a first-or second-degree relative in the same parental lineage with any cancer diagnosed under the age of 45 or a sarcoma at any age^43^. In the eight patients with a potential germline alteration of *TP53*, we found no family history of cancer in agreement with the absence of the mutations in the germline. One of these patients had a previous brain tumor (medulloblastoma) and an excessive toxicity to the chemotherapy, indicating the possibility of having a predisposition cancer syndrome, but we did not find mutations in the germline. Perhaps, this patient has another *TP53* alteration that we missed with panel sequencing. It is important to consider that the panel does not cover the whole intronic sequences. False negative results can occur because tumor-first NGS is not designed to detect germline findings but it is a complementary way to increase the detection of germline pathogenic variants in patients and their families. Therefore, tumor sequencing is not sufficient to rule out a cancer predisposition syndrome and it should be considered in the context of other criteria. The weakness of our study is that the germline was not broadly assessed for variants in a large number of cancer predisposition genes not included in the panel. The case of the patient with the Rothmund-Thomson syndrome is an example of this fact since *RECQL4* is not assayed in the panel.

In conclusion, we have identified two patients with pathogenic germline variants using the previous results of tumor sequencing and applying different criteria, confirming that somatic testing is an important source of germline findings. The early diagnosis of germline variants in pediatric cancer patients is needed to prioritize the use of inhibitors or targeted therapies for those patients who do not respond well to therapy. Identifying pathogenic variants in germline has a great impact for the patient and their families in terms of diagnosis, therapy, survival and identification of at-risk relatives. Both patients and family members affected by these syndromes require appropriate and expert genetic counseling. In the case of the centers involved in this study, there are genetic counseling units with geneticists and cancer oncologists, where the relevant tests are requested from family members and, once the results are known, genetic counseling is provided. Finding germline variants will also have an impact on the healthcare system by developing and validating genomic tools for the detection of genetic predisposition to cancer syndromes and facilitating the implementation of guidelines to improve the preventive measures and interdisciplinary care required by patients and families affected by these syndromes. In this sense, our group is taking part into a national project that aims to implement personalized medicine in children and adolescents with cancer (SEHOP-PENCIL study), which makes easier an early identification and intervention on cancer predisposition syndromes by using different NGS technologies.

NGS panels have some limitations as pathogenic mutations in non-exonic regions (promoter or deep intronic mutations) cannot be detected with panel sequencing and the pathogenic variant responsible for the syndrome can be in a gene not included in the panel. This may be addressed by incorporating additional technologies such as Whole Exome Sequencing (WES), Whole Genome Sequencing (WGS) or RNA sequencing^44–46^. In addition, these techniques make it possible to find new mutations or genes predisposing to cancer. Although germline alterations in *TP53* are the most common, new genes of equal importance are appearing over time as we have seen in our study. However, both WGS and WES have incomplete coverage^47^. Gene panels are generally designed to ensure good coverage of the genes selected and all regions of interest are well tested^48^, whereas WES and WGS have less depth of sequencing and present a higher risk of obtaining uncertain, secondary, or incidental findings that may be not related to the presentation triggering the genetic testing^47^.

It has been recently proposed that the potential for cancer predisposition should be considered for every child with cancer^3^. Although disease- and family history-based testing guidelines are useful in detecting children with underlying predisposition, it is necessary to recognize that a proportion of germline mutations will not be detected based on these guidelines and will be missed if analysis is restricted to only those meeting the criteria. However, there are ethical considerations surrounding germline sequencing of children with cancer that have to be taken into account. NGS testing has many ethical questions and concerns. If a child with cancer undergoes germline sequencing such as WES, incidental findings may be discovered. NGS testing may also reveal VUS that are not well understood. It can be difficult for families to comprehend that NGS tests may actually obtain uncertain information. Patients may be exposed to medical screenings or other procedures that can ultimately be proven unnecessary^5^.

Review of tumor data NGS increases the discovery of germline pathogenic variants from tumor testing as we have shown in pediatric sarcomas. Identifying a cancer predisposition syndrome has a huge impact in the clinical management of pediatric cancer patients and their families. The identification of patients with genetic predisposition syndromes to cancer will not only allow follow-up to be better adjusted to their real risk, but will also allow family genetic counselling, identifying other potentially young people who may benefit from predictive tests when they have not yet developed a tumor. In addition, a diagnosis of a genetic predisposition syndrome in a patient leads to changes in the treatment of the tumor, quality of life and lifelong follow-up. Thus, the identification of a genetic predisposition to cancer syndrome has an impact on diagnosis, therapy, survival and identification of family members at risk.

## Materials and methods

### Study population

The study was conducted on a cohort of 43 pediatric and young adult patients suffering from different sarcoma subtypes including osteosarcoma (n = 18), Ewing sarcoma (n = 12), rhabdomyosarcoma (n = 4) and other types (n = 9), who had surgical resection or biopsy at Hospital Universitario de Cruces (Barakaldo, Spain) and Clínica Universidad de Navarra (CUN) (Pamplona, Spain) between 2013 and 2021 (see Table 1).

This study was in accordance with international Good Clinical Practice guidelines, the Declaration of Helsinki and the national and international rules and regulations. The Law 14/2007 on Biomedical Research and Organic Law 03/2018 of 5 December on the Protection of Personal. The ethical approval was granted from Research Ethics Committee at Cruces University Hospital (E17/58) and Research Ethics Committee at University Clinic of Navarra (2017.109). All patients and/or legal guardians signed an informed consent to participate in the research. All the samples were initially processed and stored until analysis in the Basque Biobank for Research-OEHUN in accordance with the ethical principles stipulated for research with human beings.

All sarcomas included in this study were pathologically evaluated on hematoxylin–eosin stained slides, and Fluorescence In Situ Hybridization (FISH) and immunohistochemistry (IHC) were used as primary detection approaches for the possible fusion events. All these tests were performed by experienced clinical pathologists, according to the routine diagnostic procedures and laboratory standard guidelines with validated reagents. Moreover, diagnosis of different sarcoma subtypes was confirmed by a reference pathologist when required.

### Nucleic acid extraction and quantification

DNA and RNA extractions from tumor samples were carried out using Maxwell RSC DNA FFPE kit (Promega, AS1450), Maxwell RSC Tissue DNA Kit (Promega, AS1610), Maxwell RSC RNA FFPE kit (Promega, AS1440) and Maxwell RSC Simply RNA tissue (Promega, AS1340). DNA and RNA were quantified using a Qubit fluorometer and adjusted to a final quantity of 50 ng of both DNA and RNA. Complementary DNA (cDNA) was obtained using SuperScript VILO Reverse Transcriptase (Thermo Fisher Scientific).

### NGS Library Preparation and Sequencing

Tumor profiling to detect sequence alterations and abnormal gene fusions was undertaken using the Oncomine Childhood Cancer Research Assay (Thermo Fisher Scientific) according to the manufacturer’s protocol. This tool analyzes the mutational state of 203 genes including 82 mutation hotspots, 24 CNVs (copy number variants) targets, 44 genes with full exome coverage (specifically tumor suppressor genes) and a RNA panel for 97 genes (with > 1700 fusion isoform variants).

DNA and RNA libraries were generated using Ion AmpliSeq Library Preparation on the Ion chef System (Thermo Fisher Scientific). Sequencing was performed using 540 chips on the Ion Torrent GeneStudio S5 Prime (Thermo Fisher Scientific).

### Data analysis

Variants were identified and annotated with the Thermo Fisher Ion Reporter, Oncomine Knowledge Reporter and with independent manual supervision from two experts. A filter was included in the bioinformatic analysis to ensure the quality of the generated data (Q > 30). Different checkpoints were included throughout the analysis process based on the uniformity of the number of reads between samples, alignment percentages or PCR duplicate control. This analysis confirms that the data have the appropriate homogeneity, depth and complexity to use in a clinical context.

Variant calling was based on the genome version GRCh37 (hg19). A variant was primarily accepted if it was covered with at least 500 reads and tumor VAF was upper than 0.05. A CNV variant was included if the confidence at 5% was higher than or equal to 4 copies. Fusion genes with more than 50 reads were accepted. Variants were classified according to international recommendations^49,50^ as pathogenic, likely pathogenic, benign, likely benign or of uncertain significance based on literature and specific databases (Varsome, ClinVar, OncoKB, COSMIC, PeCan, TumorFusions, PanDrugs). Variants were visually inspected by using the Integrated Genomics Viewer (IGV) software^51,52^. Mutations were represented using OncoPrinter tool from cBioportal v5.1.6 (https://www.cbioportal.org/oncoprinter)^53^.

### Case selection

Tumor-first NGS results were reviewed for potential germline alterations in clinically relevant genes associated with cancer predisposition syndromes: *AKT1, ALK, APC, BRAF, CBL, CDK4, CDKN2A, CEBPA, CREBBP, DICER1, EGFR, EP300, ETV6, GATA1, GATA2, HRAS, KRAS, MAP2K1, MAP2K2, MDM2, MET, MPL, MSH6, MYC, MYCN, NF1, NF2, NPM1, NRAS, PAX5, PTCH1, PTEN, PTPN11, RAF1, RB1, RET, RUNX1, SH2B3, SH2D1A, SMARCA4, SMARCB1, SUFU, TERT, TP53, TPMT, TSC1, TSC2* and *WT1* (Table 4). The association of these genes with cancer predisposition syndromes was obtained from different databases as Genetics Home Reference and GeneCards and several studies^1,2^. The rules applied to tumor-first NGS review to identify potential germline alterations are described in Table 4. We used the 20% VAF threshold in tumor sequencing data to select potentially germline variants as it has been done in other studies and guidelines where they use a similar threshold^54,55^. Jongmans´ criteria^56^ were taken into consideration for the patient selection including family history, tumor characteristics and patient data (Table 5).

**Table 5:** Jongmans’ criteria^56^.

Family history	2 or more malignancies in family members before age 18 years, including index patient
	Parent or sibling with current or past history of cancer before age 45 years
	2 or more first or second-degree relatives in the same parental lineage with cancer before age 45 years
	Consanguinity between parents of the affected child
Tumor characteristics	Diagnosis of a type of tumor which is characteristic of a cancer predisposition syndrome
	Patient with 2 or more neoplasms, one of them before the age 18 (excluding second tumors consistent in time and/or histological type with those expected by the treatment received)
Patient data	Phenotype compatible with a cancer predisposition syndrome
	Patients with excessive toxicity to cancer treatment (toxicity not expected in type, degree or duration for the treatment received)

### Germline analysis

Genomic DNA was obtained from peripheral EDTA-blood using the FlexiGene DNA Kit (Qiagen Iberia, Madrid, Spain) and Maxwell RSC Blood DNA Kit (Promega Biotech Iberica, Madrid, Spain) following the manufacturer’s instructions. Selected variants were validated by conventional Sanger sequencing using an ABI3130xl Genetic Analyzer (Thermo Fisher Scientific).

## Supplementary Information

Supplementary Information.

## Data Availability

The datasets generated and/or analysed during the current study are available in the NCBI Sequence Read Archive repository [Accession Numbers: SRP408659 and SRR12651221].
