# Supplementary material for: Identification of germline cancer predisposition variants in pediatric sarcoma patients from somatic tumor testing
Source: Sci Rep. 2023 Feb 20;13:2959. doi: 10.1038/s41598-023-29982-2 (PMC9941115; doi:10.1038/s41598-023-29982-2)
Supplement: Supplementary file 1 — Supplementary Information. [file 41598_2023_29982_MOESM1_ESM.pdf]

Identification of germline cancer predisposition variants in pediatric sarcoma patients from somatic tumor testing

Piedad Alba-Pavón, Lide Alaña, Miriam Gutierrez-Jimeno, Susana García-Obregón, Teresa Imízcoz, Elena Panizo, Paula González-Urdiales, Aizpea Echebarria-Barona, Ricardo Lopez Almaraz, Laura Zaldumbide, Itziar Astigarraga, Ana Patiño-García and Olatz Villate

Supplementary Table S1. Potentially significant variants identified in the studied patients

PATIENT	TUMOR TYPE	AGE DIAGNOSIS	GENE	FUNCTION[a]	VARIANT NT	VARIANT AA	VAF (%)[b]	TUMOR CELLS PERCENTAGE[c]	CLINICAL SIGNIFICANCE[d]	GERMINAL CANDIDATE GENE	SUPECTED SYNDROME	GERMLINE TESTING	COMMENTS
1	Angiomatoid fibrous histiocytoma	11.4	*CIC*	TS	c.4534C>T	p.(Arg1512Cys)	5	UNK	V	NO			
2	Condrosarcoma	11.6	*IDH1*	O	c.395G>A	p.(Arg132His)	13	UNK	LP	NO			
3	Ewing sarcoma	7.4	***TP53***	**TS**	**c.404del**	**p.(Arg135SerfsTer73)**	**50**	**70%**	**P**	**YES**	**Li Fraumeni syndrome**	**Not tested**	**Unavailability of sample**
			TP53	**TS**	**c.817C>T**	**p.(Arg273Cys)**	**50**		**P**	**YES**	**Li Fraumeni syndrome**	**Not tested**	**Unavailability of sample**
			NOTCH1	TS, O	c.5177_5178insC	p.(Glu1727GlyfsTer45)	3		P	NO			
4	Ewing sarcoma	22.9	*JAK1*	O	c.2990G>A	p.(Arg997Gln)	40	>50%	V	NO			
5	Ewing sarcoma	12.5	*TSC2*	TS	c.2434A>G	p.(Ser812Gly)	50	UNK	V	NO			
6	Ewing sarcoma (Askin tumor)	6.2	***TP53***	**TS**	**c.527G>T**	**p.(Cys176Phe)**	**13**	**UNK**	**LP**	**YES**	**Li Fraumeni syndrome**		

6-Relapse	Ewing sarcoma (relapse)							UNK					
7	Ewing sarcoma	1.6	*MSH6*	TS	c.3299C>T	p.(Thr1100Met)	51	UNK	V	YES	Turcot (MB)/ Lynch syndrome		
			FLT3	O	c.950T>A	p.(Val317Glu)	49		V	NO			
8	Ewing sarcoma	10.2	*ARID1B*	TS	c.831del	p.(Glu278ArgfsTer142)	97	UNK	LP	NO			
			CDKN2A	**TS**	**c.329G>A**	**p.(Trp110Ter)**	**50**		**P**	**YES**	**Familial melanoma**	**Variant absent**	
			CRLF2	O	c.739C>T	p.(Leu247Phe)	6		V	NO			
9	Ewing sarcoma	1.5	*CRLF1*	O	c.925G>A	p.(Val309Met)	5	UNK	V	NO			
10	Ewing sarcoma	14.4	*ERBB3*	O	c.3202G>T	p.(Glu1068Ter)	52	>70%	P	NO			
			JAK2	O	c.3188G>A	p.(Arg1063His)	50		V	NO			
			WT1	TS	c.1048T>C	p.(Cys350Arg)	46		V	NO			
11	Ewing sarcoma	14.3	*CIC*	TS	c.4534C>T	p.(Arg1512Cys)	12	UNK	V	NO			
12	Myxoid liposarcoma	9.3	*PTEN*	TS	c.961del	p.(Thr321GlnfsTer23)	20	>90%	P	NO			
			PIK3CA	O	c.3140A>G	p.(His1047Arg)	60		P	NO			
13	Malignant peripheral nerve sheath tumor (MPNST)	12.3	*SUZ12*	TS	c.2021_2028del	p.(Ile674SerfsTer7)	90	UNK	P	NO			
			NF1	**TS**	**c.7152_7153insT**	**p.(Asn2385Ter)**	**50**		**P**	**YES**	**Neurofibromatosis**	**Variant detected**	
14	Osteosarcoma	15.2	***TP53***	**TS**	**c.957_958insC**	**p.(Lys320GlnfsTer17)**	**60**	**UNK**	**P**	**YES**	**Li Fraumeni syndrome**	**Variant absent**	
15	Osteosarcoma	11.7	***CDKN2A***	**TS**	**c.350del**	**p.(Leu117ArgfsTer29)**	**84**	**UNK**	**P**	**YES**	**Familial melanoma**	**Variant detected**	
			TET2	TS	c.344A>G	p.(Asp115Gly)	12		V	NO			

			TET2	TS	c.3969G>C	p.(Glu1323Asp)	9		V	NO			
16	Osteosarcoma	6.1	***TP53***	**TS**	**c.818G>A**	**p.(Arg273His)**	**85**	**UNK**	**P**	**YES**	**Li Fraumeni syndrome**	**Variant absent**	
17	Osteosarcoma	13.5	***TP53***	**TS**	**c.818G>A**	**p.(Arg273His)**	**64**	**UNK**	**P**	**YES**	**Li Fraumeni syndrome**	**Variant absent**	
			TSC1	TS	c.640G>T	p.(Glu214Ter)	47		LP	NO			
			ARID1B	TS	c.3751C>T	p.(Arg1251Trp)	82		V	NO			
			CRLF2	O	c.406G>A	p.(Val136Met)	54		V	NO			
18	Osteosarcoma	2.6	***RB1***	**TS**	**c.1150C>T**	**p.(Gln384Ter)**	**13**	**UNK**	**P**	**YES**	**Hereditary Retinoblasto ma**	**Not tested**	
			TP53	**TS**	**c.475G>C**	**p.(Ala159Pro)**	**28**		**P**	**YES**	**Li Fraumeni syndrome**	**Variant absent**	
			JAK3	O	c.2773C>A	p.(Arg925Ser)	50		V	NO			
19	Osteosarcoma (relapse)	16.1	*ARID1A*	TS	c.2951A>G	p.(Lys984Arg)	77	UNK	V	NO			
20	Osteosarcoma	7.4	***RB1***	**TS**	**c.1215+1G>A**	**p.?**	**65**	**UNK**	**P**	**YES**	**Hereditary Retinoblasto ma**	**Variant absent**	
21	Osteosarcoma	7.1	*CEBPA*	TS	c.568T>C	p.(Ser190Pro)	62	UNK	V	NO			
22	Osteosarcoma	10.2	*KMT2D*	TS	c.7478G>T	p.(Gly2493Val)	50	>70%	V	NO			
			WT1	TS	c.791C>G	p.(Pro264Arg)	38		V	YES			
			APC	TS	c.3395A>G	p.(Glu1132Gly)	5		V	YES			
23	Osteosarcoma (3rd relapse)	15.4	*RUNX1*	TS	c.1113G>A	p.(Met371Ile)	24		V	YES	Platelet disorder associated with Mieloid Malignancies		
			ARID1A	TS	c.388G>A	p.(Asp130Asn)	29		V	NO			
			CIC	TS	c.4533_4534delin sTT	p.(Arg1512Cys)	13		V	NO			

24	Chondroblastic osteosarcoma	17.9	***TP53***	**TS**	**c.618_624del**	**p.(Asp207Glufs Ter38)**	**60**	**UNK**	**P**	**YES**	**Li Fraumeni syndrome**	**Variant absent**	
			NOTCH1	TS, O	c.7250C>T	p.(Pro2417Leu)	50		V	NO			
			KDM6A	TS	c.3578G>C	p.(Trp1193Ser)	7		V	NO			
			ASXL1	TS	c.1831G>A	p.(Ala611Thr)	50		V	NO			
			KMT2D	TS	c.8432A>G	p.(Gln811Arg)	50		V	NO			
			SMARCA4	TS	c.4813G>A	p.(Glu1605Lys)	50		V	YES	Rhabdoid tumor predisposition syndrome	Not tested	
25	Chondroblastic osteosarcoma	12.7	*DICER1*	TS	c.2243G>A	p.(Cys748Tyr)	30	50%	V	YES			
25-Metastasis	Costal metastasis of primary osteosarcoma		***RB1***	**TS**	**c.1422-1GC>TT**	**p.?**	**70**		**P**	**YES**	**Hereditary Retinoblasto ma**		
26	Osteoblastic osteosarcoma	22.8	*CHD7*		c.1397C>T	p.(Ser466Leu)	50	>90%	V	NO			
27	Osteoblastic osteosarcoma	5.1	***RB1***	**TS**	**c.1654C>T**	**p.(Arg552Ter)**	**30**	**100%**	**P**	**YES**	**Hereditary Retinoblasto ma**		**Child with congenital or cancer syndromes Rothmund Thompson syndrome**
28	Osteo-chondroblastic osteosarcoma	15.1	*CHD7*		c.1510C>T	p.(Gln504Ter)	10	<10%	LP	NO			
29	Osteo-chondroblastic osteosarcoma	16.4	*KMT2D*	TS	c.15456C>G	p.(Phe5152Leu)	50	20%	V	NO			
30	Parostal osteosarcoma	12.7	*NF2*	TS	c.1051C>T	p.(Arg351Cys)	50	100%	V	YES	Neurofibromat osis		

31	Alveolar rhabdomyosarcoma	18.1	*JAK1*	O	c.3344C>T	p.(Pro1115Leu)	50	>50%	V	NO			
32	Alveolar rhabdomyosarcoma	13.3	*ASXL2*	**TS**	c.1367_1368insA	p.(Asn456LysfsTer18)	90	<50%	P	NO			
			TP53	**TS**	**c.817C>T**	**p.(Arg273Cys)**	**60**		**P**	**YES**	**Li Fraumeni syndrome**	**Variant absent**	
			NF2	**TS**	**c.586C>T**	**p.(Arg196Ter)**	**60**		**P**	**YES**	**Neurofibromatosis**	**Variant absent**	
33	Embryonal rhabdomyosarcoma	8.8	***SMARCA4***	**TS**	**c.3694G>A**	**p.(Gly1232Ser)**	**20**	**>90%**	**P**	**YES**	**Rhabdoid tumor predisposition syndrome**	**Not tested**	**Unavailability of sample**
34	Embryonal rhabdomyosarcoma	6.4	***TP53***	**TS**	**c.438_439insGTTGATTCCACACCCCC**	**p.(Pro153LeufsTer23)**	**10**	**UNK**	**P**	**NO**	**Li Fraumeni syndrome**		
			TP53	**TS**	**c.524G>A**	**p.(Arg175His)**	**18**		**P**	**NO**	**Li Fraumeni syndrome**		
			MDM2	TS	c.499A>G	p.(Arg167Gly)	52		V	NO			
35	Undifferentiated sarcoma	17.5	*PIK3CA*	O	c.1633G>A	p.(Glu545Lys)	30	UNK	P	NO			
35-Relapse	Undifferentiated sarcoma (relapse)							UNK					
36	Synovial sarcoma	10.9	*RB1*	TS	c.1093G>C	p.(Glu365Gln)	42	UNK	V	NO			
			FAS	TS	c.580G>A	p.(Glu194Lys)	49		V	NO			
37	Solitary fibrous tumor	30.8	***TP53***	**TS**	**c.713G>A**	**p.(Cys238Tyr)**	**40**	**70%**	**P**	**YES**	**Li Fraumeni syndrome**	**Not tested**	**Unavailability of sample**
38	Soft tissue rhabdoid tumor (relapse)	4.3	*KMT2D*	TS	c.6554_6555insC	p.(Glu2186GlyfsTer16)	6	UNK	P	NO			

			SMARCB1	**TS**	**c.544C>T**	**p.(Gln182Ter)**	**95**		**LP**	**YES**	**Rhabdoid tumor predisposition syndrome**	**Variant absent**	
			ABL1	O	c.995G>A	p.(Arg332Gln)	51		V	NO			
39	Soft tissue rhabdoid tumor	0.6	***SMARCB1***	**TS**	**c.472C>T**	**p.(Arg158Ter)**	**42**	**80%**	**P**	**YES**	**Rhabdoid tumor predisposition syndrome**	**Variant absent**	
			SMARCB1	**TS**	**c.118C>T**	**p.(Arg40Ter)**	**42**		**P**	**YES**	**Rhabdoid tumor predisposition syndrome**	**Variant absent**	
40	Osteosarcoma	9.9	*KMT2D*	TS	c.5717A>G	p.His1906Arg	52	>60%	V	NO			
			PHF6	TS	c.1045A>G	p.Lys349Glu	97		V	NO			
			FGFR4	O	c.1703C>A	p.Pro568Gln	40		V	NO			
41	Ewing's sarcoma	13.9	***CDKN2A***	**TS**	**c.172C>T**	**p.Arg58Ter**	**11**	**>60%**	**P**	**YES**	**Familial Melanoma**		
42	Ewing's sarcoma	10.9	*APC*	TS	c.3949G>C	p.Glu1317Gln	53	>60%	V	YES	Familial adenomatous polyposis		
43	Ewing's sarcoma (relapse)	14.5	*CCND3*	O	c.857A>T	p.Asp286Val	18	>60%	V	NO			

[a]TS: tumor suppressor; O: oncogene;[b]VAF: variant allele frequency [c]UNK: unknown; [d]P: Pathogenic; LP: likely pathogenic; V: variant of unknown clinical significance, VUS.

Germline candidate genes with pathogenic or likely pathogenic variants are highlighted in bold. Cases selected with a potentially germline variant are indicate in grey.
